# Combination Therapy of Lenvatinib and Hepatic Arterial Infusion Chemotherapy Using Cisplatin With Lipiodol and 5-Fluorouracil: A Potential Breakthrough Therapy for Unresectable Advanced Hepatocellular Carcinoma

**DOI:** 10.7759/cureus.66185

**Published:** 2024-08-05

**Authors:** Susumu Maruta, Yohei Koshima, Takahiro Tsuchiya, Ryo Tamura, Masanori Takahashi, Tadashi Ohshima, Yoshihiko Ooka

**Affiliations:** 1 Department of Hepatobiliary and Pancreatic Oncology, Saitama Red Cross Hospital, Saitama, JPN; 2 Department of Digestive Internal Medicine, Saitama Red Cross Hospital, Saitama, JPN; 3 Department of Internal Medicine, Chiba Chuo Clinic, Chiba, JPN

**Keywords:** len-new fp, bevacizumab, atezolizumab, new fp, hepatic arterial infusion chemotherapy, lenvatinib, hepatocellular carcinoma

## Abstract

Introduction: In 2021, the LEOPARD trial reported that the combination of lenvatinib+one-shot cisplatin infusion might contribute to improving the results of conventional advanced hepatocellular carcinoma (HCC) treatment. Thus, combination therapy with lenvatinib and catheterization has emerged as a focal point in treating advanced HCC. Conversely, the New FP regimen consists of low-dose cisplatin (CDDP) combined with 5-fluorouracil (5-FU) and lipiodol via hepatic arterial infusion chemotherapy (HAIC), with a high response rate of approximately 70%. Therefore, lenvatinib+New FP (LEN-New FP) may be a more promising treatment for HCC. Here, we report six patients who were administered LEN+New FP and achieved high therapeutic efficacy. Among them, one case had an interesting clinical course, which has been described in detail.

Materials and methods: This study included six patients who were administered 12 mg or 8 mg of lenvatinib once daily based on a body weight of ≥60 kg or <60 kg, respectively, along with 50 mg of cisplatin in 5-10 mL lipiodol, and a continuous infusion of 5-FU (1500 mg/5 days) infused every 2-4 weeks. Tumor evaluations were performed 4-8 weeks after the initiation of New FP administration and every 8-12 weeks thereafter.

Results: The median patient age was 65 years. All patients had a history of prior treatment with atezolizumab and bevacizumab and one of the factors associated with poor overall survival for New FP monotherapy, such as a maximum tumor diameter ≥7 cm and bilobular multifocal distribution. Four (67%) patients had severe vascular invasion. The best objective response and disease control rates were 83% and 100%, respectively. The best response of the target lesion was complete remission in four out of six patients.

Conclusion: The LEN-New FP combination for advanced HCC showed a high response rate and was more effective in high-risk patients with factors associated with poor overall survival than that reported with conventional New FP monotherapy. Additionally, LEN-New FP exhibited extremely high objective response and disease control rates and was well tolerated, including in cases where it was considered second- or third-line systemic chemotherapy for advanced HCC. Thus, LEN-New FP can serve as a breakthrough therapy for advanced HCC based on appropriate case selection.

## Introduction

Current state of treatment for advanced hepatocellular carcinoma (HCC)

In June 2009, molecularly targeted agents (MTAs), such as sorafenib, were shown to prolong survival in patients with advanced HCC for the first time; systemic therapy for HCC has changed dramatically since then [1,2]. With the advent of new treatment options for advanced HCC, patients now have the potential for some degree of long-term survival. Sorafenib, despite its lack of tumor reduction capabilities and characteristic adverse events like hand-foot skin reactions, paved the way for several novel MTAs including brivanib, everolimus, and tivantinib. However, these drugs were unsuccessful in clinical trials, marking a challenging period in the development of systemic treatments post-sorafenib [3]. Nevertheless, in 2017, regorafenib emerged as a second-line treatment for HCC [4]. Additionally, lenvatinib has been recognized as non-inferior to sorafenib for first-line HCC treatment since 2018 [5]. More recently, immune checkpoint inhibitors (ICIs), such as atezolizumab plus bevacizumab [6] and durvalumab plus tremelimumab [7], have received approval for HCC treatment, representing significant advancements in systemic therapy for advanced HCC that continues to evolve.

Beneath the brilliant development of systemic therapy underlies hepatic arterial infusion chemotherapy (HAIC), a transvascular treatment, with a history of use in the treatment of advanced HCC, mainly in Japan. However, insufficient evidence and a lack of uniformity in regimens remain persistent problem areas. In addition, the international position of HAIC in the treatment of advanced HCC remains undetermined.

Current status of HAIC

HAIC has been actively used since the 1990s, mainly in Japan. It enables an increased drug concentration to be administered to the liver by inserting a port catheter into the hepatic artery and administering a reduced volume of anticancer drugs, thereby enhancing the antitumor effect. This approach is considered advantageous owing to its high response rate and reduced incidence of side effects, given the minimal amount of anticancer drug used and its metabolism in the liver before systemic distribution. In daily clinical practice, the effectiveness of HAIC is particularly noted in advanced HCC, especially in cases with vascular invasion. This is reflected in the 2023 edition of the Japan Society of Hepatology treatment algorithm, according to which transcatheter arterial chemoembolization (TACE), HAIC, and molecularly targeted drugs are recommended for cases without vascular invasion and with four or more tumors [8]. However, limited large-scale, high-evidence, randomized controlled trials (RCTs) have focused on HAIC, a transvascular treatment that necessitates systemic chemotherapy and requires specialized techniques for catheter placement. This is because setting up RCTs that compare one treatment with another is challenging, unlike RCTs that compare drug treatments. Furthermore, the regimen for HAIC is not uniform and includes a variety of options, such as low-dose cisplatin (CDDP) combined with 5-fluorouracil (5-FU), known as low-dose FP [9]; CDDP, lipiodol, and 5-FU, known as New FP [10]; and FOLFOX-HAIC, which includes fluorouracil, leucovorin, and oxaliplatin [11]. Therefore, the European Association for the Study of the Liver guidelines do not endorse HAIC as a recommended treatment but suggest TACE for patients with intermediate stage B and advanced stage C disease, who have either extrahepatic metastases or vascular invasion and for whom systemic chemotherapy remains the sole recommended option [12]. In this context, it should be considered that HAIC is currently recognized around the world as a treatment that is only available in Asia. Specifically, since a catheter is inserted into the hepatic artery for direct chemotherapy administration, there is a risk that HCC, similar to TACE, possibly nourished by an artery outside the liver, may recur and anticancer drugs might not be effective against extrahepatic lesions. Moreover, Niizeki et al. [13] found that tumors >7 cm in maximum size (though not significant in multivariate analysis, but significant in univariate analysis) and tumors spread across three or more regions (i.e., multiple HCC in both lobes) significantly correlated with a poorer prognosis following HAIC.

Combination therapy of lenvatinib and HAIC using New FP

In recent years, several clinical trials have reported the combination treatment of lenvatinib and TACE, with the concept of LEN-TACE gaining popularity [14,15]. In addition, the LEOPARD trial (lenvatinib plus HAIC with cisplatin: LEOPARD, phase 2), which combined lenvatinib and a one-shot CDDP hepatic infusion [16], reported favorable results, with an objective response rate of 64.7% (95% confidence interval (CI): 46.5-80.3%)/45.7% (95% CI: 28.8-63.4%), a progression-free survival (PFS) at 6.3 months (95% CI: 5.1-7.9 months), and an overall survival (OS) at 17.2 months (95% CI: 10.9-NA months) according to the modified Response Evaluation Criteria in Solid Tumors (mRECIST)/RECISTv1.1. These treatments do not contain ICIs, enabling their use in patients with contraindications to ICIs, such as autoimmune diseases. Furthermore, these treatments are lower in cost when compared with ICIs, implying economic advantages. Thus, combination therapy with lenvatinib and catheterization has emerged as a focal point in treating advanced HCC.

Furthermore, several recent evidence-based studies have compared the safety and curative effect between TACE and HAIC for unresectable HCC, showing HAIC is more beneficial than standard TACE treatment [17,18]. Moreover, a study evaluating the New FP regimen, which differs from the lenvatinib plus one-shot CDDP infusion regimen of the LEOPARD trial, reported a high response rate of about 70% [19]. Therefore, we believe that the lenvatinib+New FP (LEN-New FP) combination may offer a more promising approach for LEN-HAIC. Here, we provide a brief summary of six patients with HCC treated with LEN-New FP with promising results and provide a detailed clinical history of one case that had an interesting course.

## Materials and methods

Participants

Patients with HCC who previously received systemic therapy (atezolizumab+bevacizumab, ±sorafenib, ±lenvatinib), HAIC, or immunotherapy and who were ineligible for resection, percutaneous ablation therapy, or TACE were included in the study. Written informed consent was obtained from the study participants. This study was approved by the Institutional Review Board of Saitama Red Cross Hospital as a retrospective clinical study (approval number: 24-J).

The following criteria were considered for the application of LEN-New FP therapy: (i) age ≥20 years, (ii) Child-Pugh class A or B, and (iii) performance status (Eastern Cooperative Oncology Group (ECOG)) level 0-2.

Cases with successful outcomes from New FP alone were not included in this study.

Catheter placement

A Shepherd hook-type 4-French catheter was inserted into the femoral artery via a 4-French introducer sheath using the Seldinger technique. The catheter was advanced to the target artery under fluoroscopy. The right gastric artery and gastroduodenal artery were placed in microcoils (Target 360 XL, Target 360 XXL, Stryker; Ruby Coil, POD packing coil, Penumbra; or VortX Diamond Shape Fibered Platinum Coils, VortX Fibered Platinum Coils, Boston Scientific) to prevent gastroduodenal damage caused by anticancer drugs. Embolization was performed using VortX Fibered Platinum Coils (Boston Scientific). We modified blood circulation to the right hepatic artery, right inferior phrenic artery, etc. as appropriate for cases with abnormal blood vessels or tumors with extrahepatic blood supply channels.

A polyurethane-covered catheter (Anthron P-U catheter, Toray Medical, Tokyo, Japan, or PIOLAX W spiral catheter, PIOLAX, Kanagawa, Japan) was used as the indwelling catheter. The gutter position of the catheter was placed in the common or proper hepatic artery. The other end of the catheter was connected to the injection port. The device was implanted in the subcutaneous pocket.

Treatment protocol

**Figure 1:**
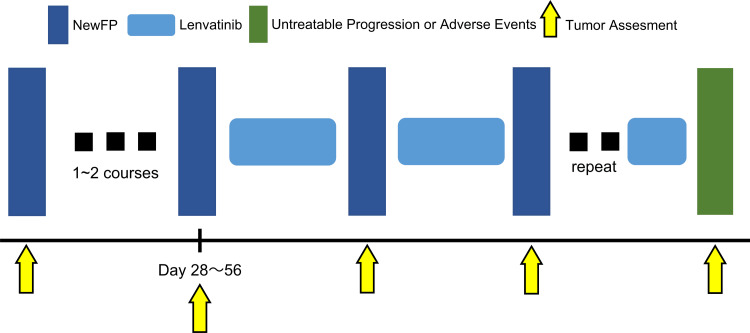
Lenvatinib was started 14-21 days before the second or third New FP cycle and continued until the event occurred. Lenvatinib administration was discontinued two days before New FP started and was resumed after New FP completion. New FP was administered regularly at 14-21-day intervals. Tumor evaluation was performed 4-8 weeks after the initial New FP cycle and every 8-12 weeks thereafter.

Lenvatinib was administered at a dose of 12 mg or 8 mg once daily to patients with a body weight ≥60 kg or <60 kg, respectively, 14-28 days after catheter placement, discontinued two days before New FP administration, and resumed two days after New FP administration (Figure 1). Considering adverse events, performance status, and age, the lenvatinib dose was reduced as required at the discretion of the attending physician. Tumor evaluations were performed 4-8 weeks after the initiation of New FP administration and every 8-12 weeks thereafter. The New FP regimen comprised 50 mg of cisplatin in 5-10 mL lipiodol and a continuous infusion of 5-FU (1500 mg/5 days) infused every 2-4 weeks in the outpatient department, for as long as possible. On the first day of treatment, cisplatin mixed with lipiodol was administered through the reservoir catheter, immediately followed by an initial dose of 5-FU (250 mg). Subsequently, 5-FU (1250 mg) was continuously infused over the next five days using a balloon pump (SUREFUSER PUMP; Nipro Pharma Corporation, Osaka, Japan). The cisplatin dose was adjusted by the angiographer each time, based on angiogram results.

Evaluation of response to chemotherapy

The primary efficacy endpoint was objective tumor response, which was initially within 4-8 weeks and subsequently every 2-3 months thereafter. Tumors were measured two-dimensionally by dynamic CT or MRI. Radiological evaluation of response to treatment was performed using mRECIST [20].

Assessment of tolerability

Safety was assessed based on adverse events, using the Common Terminology Criteria for Adverse Events version 5.0. Treatment was discontinued when adverse events reached level 2 of the ECOG classification. Complications related to indwelling catheters (e.g., gastroduodenal ulcers, infections, etc.) were also evaluated.

## Results

We administered the LEN-New FP regimen to six patients who achieved high therapeutic efficacy.

The patients with HCC treated with LEN-New FP at our hospital are presented in Table 1. The median age of the patients was 65 years, five patients were male with non-B-non-C HCC, and all patients had a Child-Pugh score of 5-6. All patients had undergone prior atezolizumab bevacizumab treatment and exhibited OS failure factors (maximum tumor size ≥7 cm and tumors involving ≥3 areas) for New FP monotherapy, as outlined by Niizeki et al. [13]. We defined these HCC conditions as the Niizeki criteria. Moreover, 67% (4/6) of the patients had severe vascular invasion. The best objective response rate was 83%. The best disease control rate reached 100%. Complete response (CR) was achieved in the target lesion in four of the six patients. Adverse events necessitated the discontinuation of treatment for two patients. Treatment was discontinued in one patient due to occlusion of the arterial infusion port catheter and in the other patient due to the onset of stroke.

**Table 1:** Six patients with LEN+New FP treated at our hospital. HCV: hepatitis C virus; AFP: alpha-fetoprotein; CR: complete response; PD: progressive disease; DCP: des-γ-carboxy prothrombin; MVI: macrovascular invasion; EHM: extrahepatic metastasis; BCLC: Barcelona clinic liver cancer; M: multiple (>7); SD: stable disease; PR: partial response

	Age	Sex	Etiology	Child-Pugh score	AFP (ng/mL)	DCP (mAU/ml)	Maximum size of intrahepatic lesions	Number of intrahepatic lesions	Tumor located segments	Niizeki criteria	Up-to-seven	MVI	EHM	BCLC stage	Best response (target lesions)	Best response (nontarget lesions)	Best response (overall response)	History of systemic chemotherapy
1	5X	Male	Non-B non-C	5	10400	59800	84	M	4	Out	Out	Vp4	＋	C	CR	Non-CR/non-PD	PR	Atezolizumab/bevacizumab
2	7X	Male	Non-B non-C	6	3.5	248	30	M	4	Out	Out	Vp4	+	C	CR	Non-CR/non-PD	PR	Sorafenib, atezolizumab/bevacizumab
3	5X	Male	Non-B non-C	6	6230	1020	127	3	3	Out	Out	Vp4	−	C	SD	Not evaluated	Not evaluated	Atezolizumab/bevacizumab
4	7X	Male	Non-B non-C	5	21.9	37	29	M	4	Out	Out	−	−	B	CR	CR	CR	Lenvatinib, atezolizumab/bevacizumab
5	6X	Male	Non-B non-C	6	10.3	305	23	M	4	Out	Out	−	−	B	CR	Non-CR/non-PD	PR	Lenvatinib, atezolizumab/bevacizumab
6	7X	Female	HCV	5	207	222	80	2	2	Out	Out	VV3	-	B	PR	Non-CR/non-PD	PR	Lenvatinib, atezolizumab/bevacizumab

One patient had an interesting clinical course wherein the combined effect of lenvatinib and New FP was easily observed.

Case

**Figure 2:**
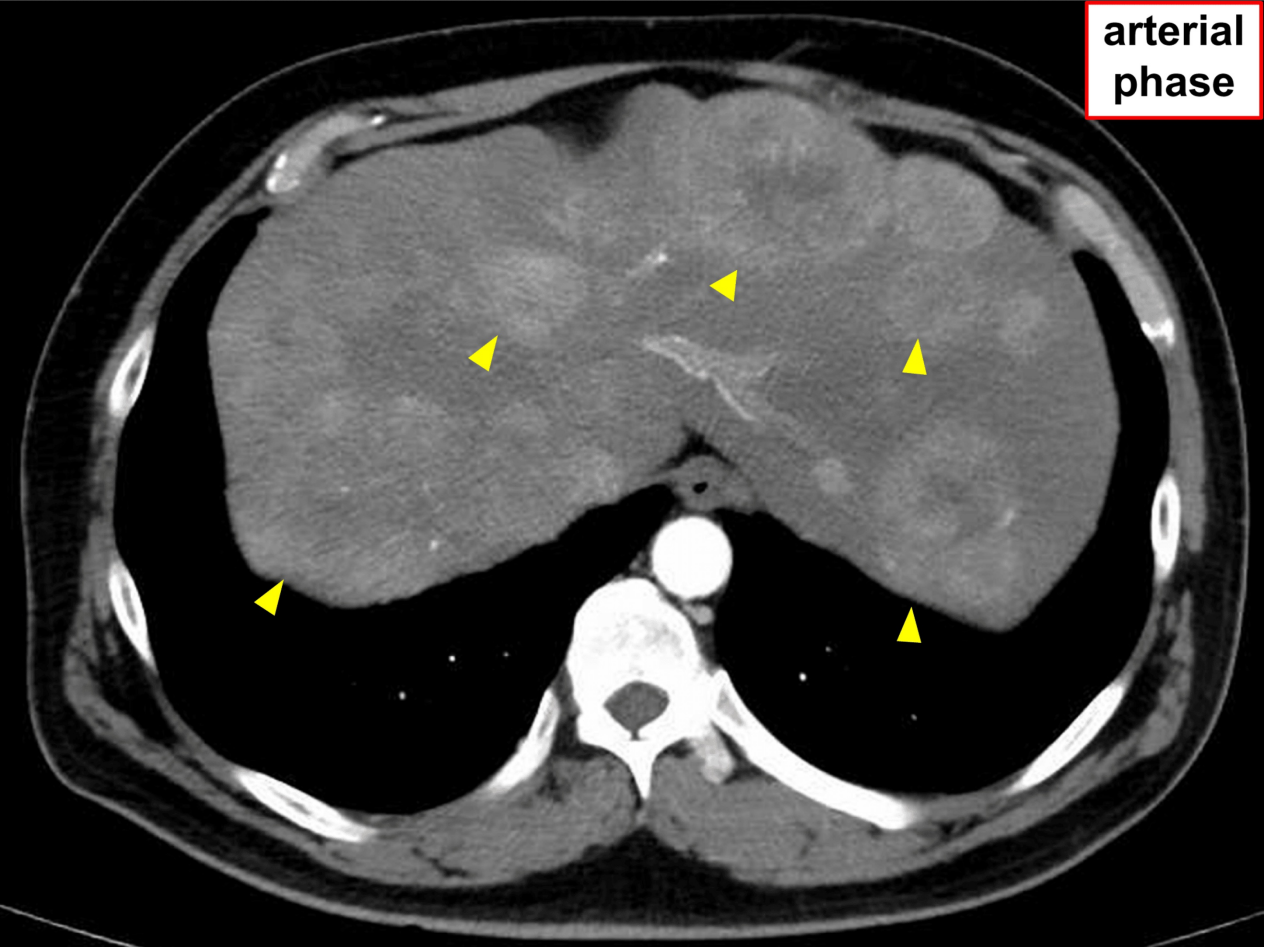
Contrast-enhanced CT tomography at the first visit. Multiple hepatic masses were observed in both liver lobes (arrowheads), which were highly stained in the arterial phase and showed washout in the equilibrium phase.

The patient was a man in his 50s, previously diagnosed with diabetes and fatty liver disease at a local clinic. In December 2021, he visited his local doctor for pain in the right hypochondrium, when an abdominal ultrasound showed multiple liver masses. The patient was admitted to our hospital, and a contrast-enhanced CT of the thoracoabdominal pelvic region was performed. Multiple hepatic masses with dark staining in the arterial phase and washout in the equilibrium phase were observed in both lobes of the liver (Figure 2). However, no clear signs of cirrhosis were observed in background liver tissues. Blood tests (Table 2) were negative for both hepatitis markers, HB antigen and hepatitis C virus antibodies, and negative for antinuclear and antimitochondrial M2 antibodies. However, tumor markers alpha-fetoprotein (AFP) (4450 ng/ml), AFPL3 (9.4%), and PIVKA-II (34500 mAU/ml) were elevated. The patient was diagnosed with HCC (intermediate stage, up to 7 points) based on liver biopsy. Treatment with atezolizumab+bevacizumab was initiated. Atezolizumab+bevacizumab therapy continued on an outpatient basis, achieving mRECIST: SD on contrast-enhanced CT scans, evaluated every 2-3 months. 

**Table 2:** Blood sampling results at the first visit. AFP: alpha-fetoprotein; AFPL3: alpha-fetoprotein L3 percent; ALP: alkaline phosphatase; ALT: alanine aminotransferase; APTT: activated partial thromboplastin time; AST: aspartate aminotransferase; BUN: blood urea nitrogen; CA19-9: carbohydrate antigen 19-9; Ch-E: cholinesterase; Cre: creatinine; CRP: C-reactive protein; D-Bil: direct bilirubin; γGTP: gamma-glutamyl transferase; Hb: hemoglobin; HbA1c: hemoglobin A1c; HBc-Ab: hepatitis B core antibody; HBs-Ab: hepatitis B surface antibody; HBs-Ag: hepatitis B surface antigen; HCV-Ab: hepatitis C virus antibody; Hct: hematocrit; LDH: lactate dehydrogenase; NH3: ammonia; PIVKA-2: protein induced by vitamin K absence or antagonist-II; Plt: platelets; PT: prothrombin time; RBC: red blood cells; T-Bil: total bilirubin; T-Chol: total cholesterol; TP: total protein; WBC: white blood cells

Test	Results	Reference values
Blood chemistry		
TP	7.3 g/dL	6.6-8.1
Alb	4.4 g/dL	4.1-5.1
T-Bil	1.0 mg/dL	0.4-1.5
D-Bil	0.3 mg/dL	0-0.4
BUN	14.3 mg/dL	8-20
Cre	0.89 mg/dL	0.65-1.07
NH_3_	30 μg/dL	4-50
AST	82 U/L	13-30
ALT	73 U/L	10-42
ALP	148 U/L	38-113
LDH	194 U/L	124-222
γGTP	112 U/L	13-64
Ch-E	310 U/L	240-486
T-Chol	148 mg/dL	142-248
CRP	0.18 mg/dL	0-0.14
HbA1c	6.2%	4.9-6.0
HBs-Ag	(−)	
HBs-Ab	(+)	
HBc-Ab	(+)	
HCV-Ab	(−)	
Peripheral blood		
RBC	535×10^４^/μl	555-435
Hb	16.1g/dL	13.7-16.8
Hct	48.2%	40.7-50.1
WBC	6100/μL	3300-8600
Plt	27.6×10^４^/μL	15.8-34.8
Coagulation		
APTT	37.1sec	27.1-39.3
PT	11.1sec	10.2-12.7
D-dimer	1.0 μg/ｍL	0-1.1
Tumor marker		
CEA	7.8 ng/mL	0-5
CA19-9	26.0 U/mL	0-37
AFP	4450 ng/ml	0-10
AFPL3	9.4%	0-10
PIVKA-2	34500 mAU/ml	0-40

**Figure 3:**
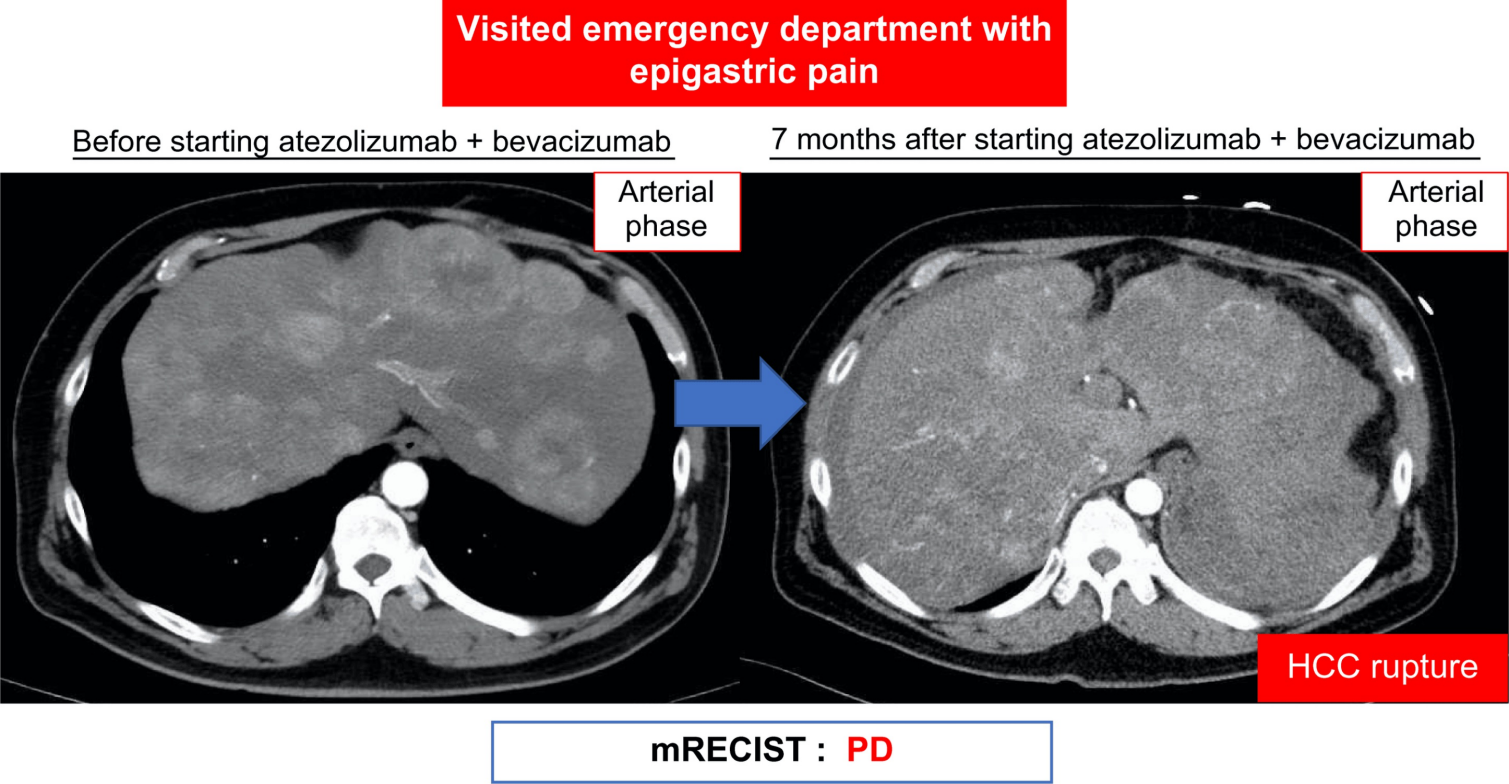
Contrast-enhanced CT tomography revealed hematoma formation around the HCC in the liver S2, leading to a diagnosis of ruptured hepatocellular carcinoma. mRECIST: modified Response Evaluation Criteria in Solid Tumors; PD: progressive disease; HCC: hepatocellular carcinoma

**Figure 4:**
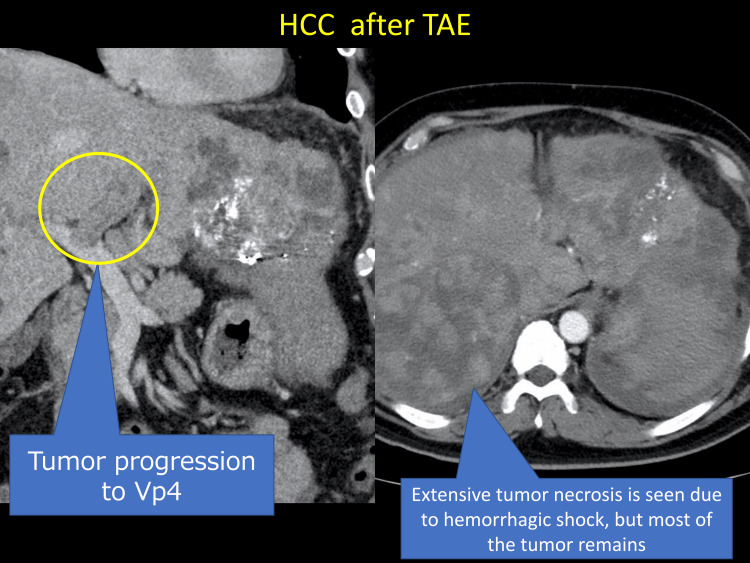
Contrast-enhanced CT after TAE for ruptured HCC. HCC: hepatocellular carcinoma; TAE: transarterial embolization

**Figure 5:**
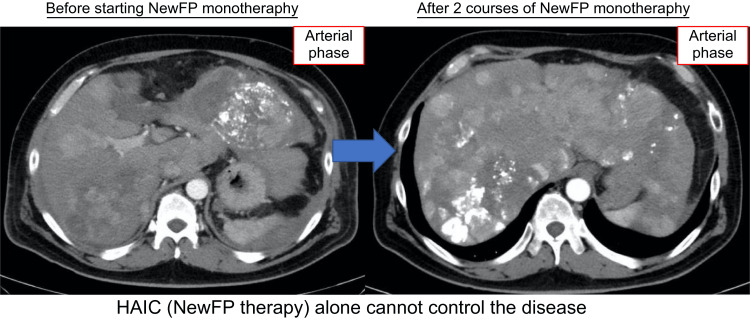
After two courses of the New FP therapy, contrast-enhanced CT evaluation revealed an increase in target lesions, the appearance of new intrahepatic lesions, and an upward trend in tumor markers. HAIC: hepatic arterial infusion chemotherapy

**Figure 6:**
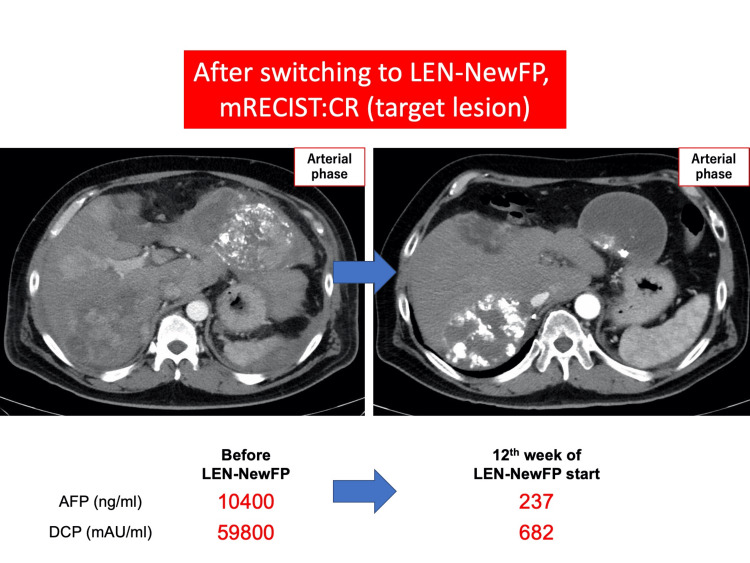
Switching from HAIC (New FP) monotherapy to LEN+New FP treatment was effective. mRECIST: modified Response Evaluation Criteria in Solid Tumors; CR: complete response; LEN: lenvatinib; AFP: alpha-fetoprotein; DCP: des-γ-carboxy prothrombin

**Figure 7:**
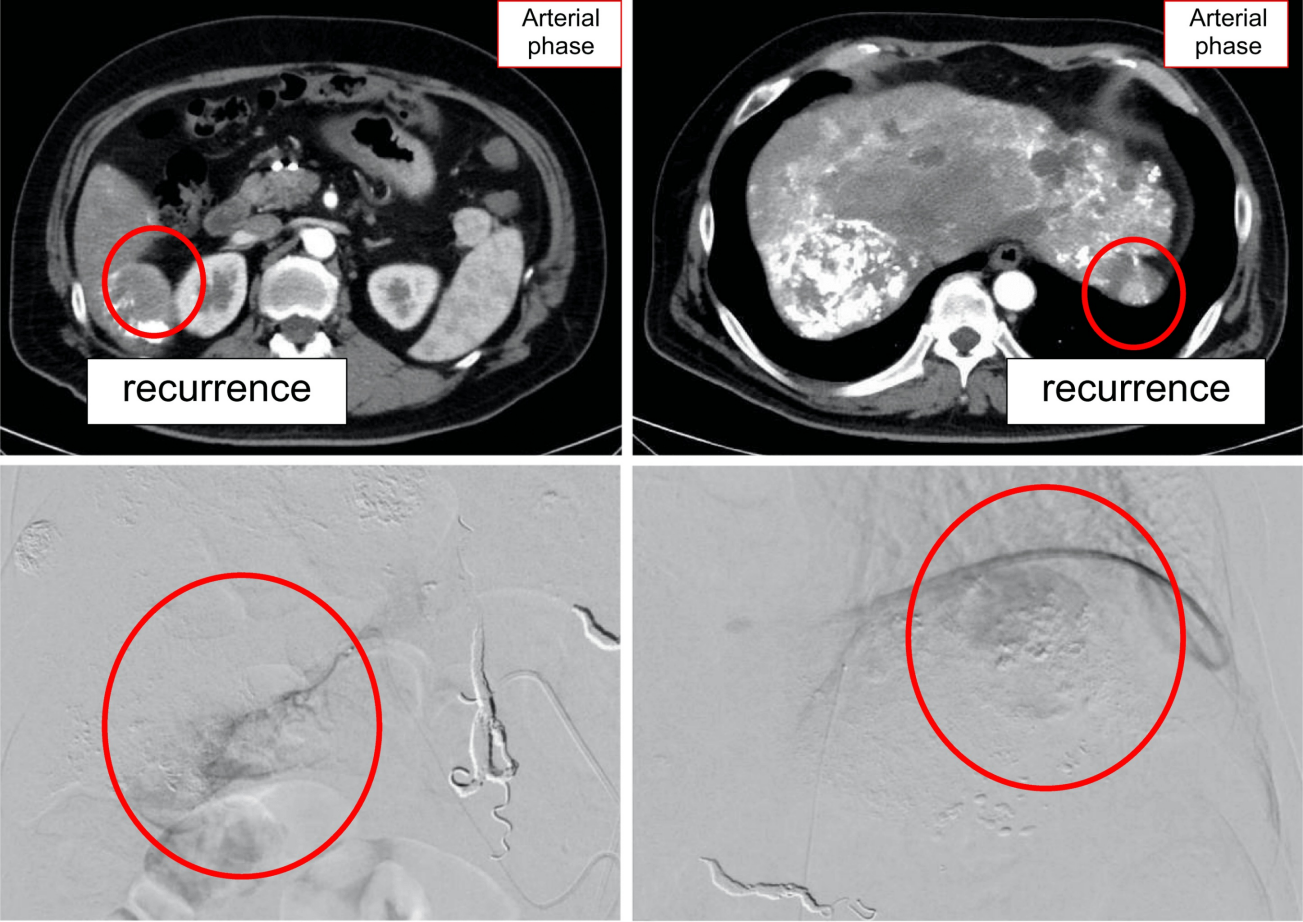
Intrahepatic tumor recurrence in the S6 and S2 liver regions. However, because the S6 tumor recurred in the right renal capsular artery and the S2 tumor recurred in the left inferior phrenic artery, TACE plus SBRT was added to this region for local control. TACE: transcatheter arterial chemoembolization; SBRT: stereotactic body radiotherapy

**Figure 8:**
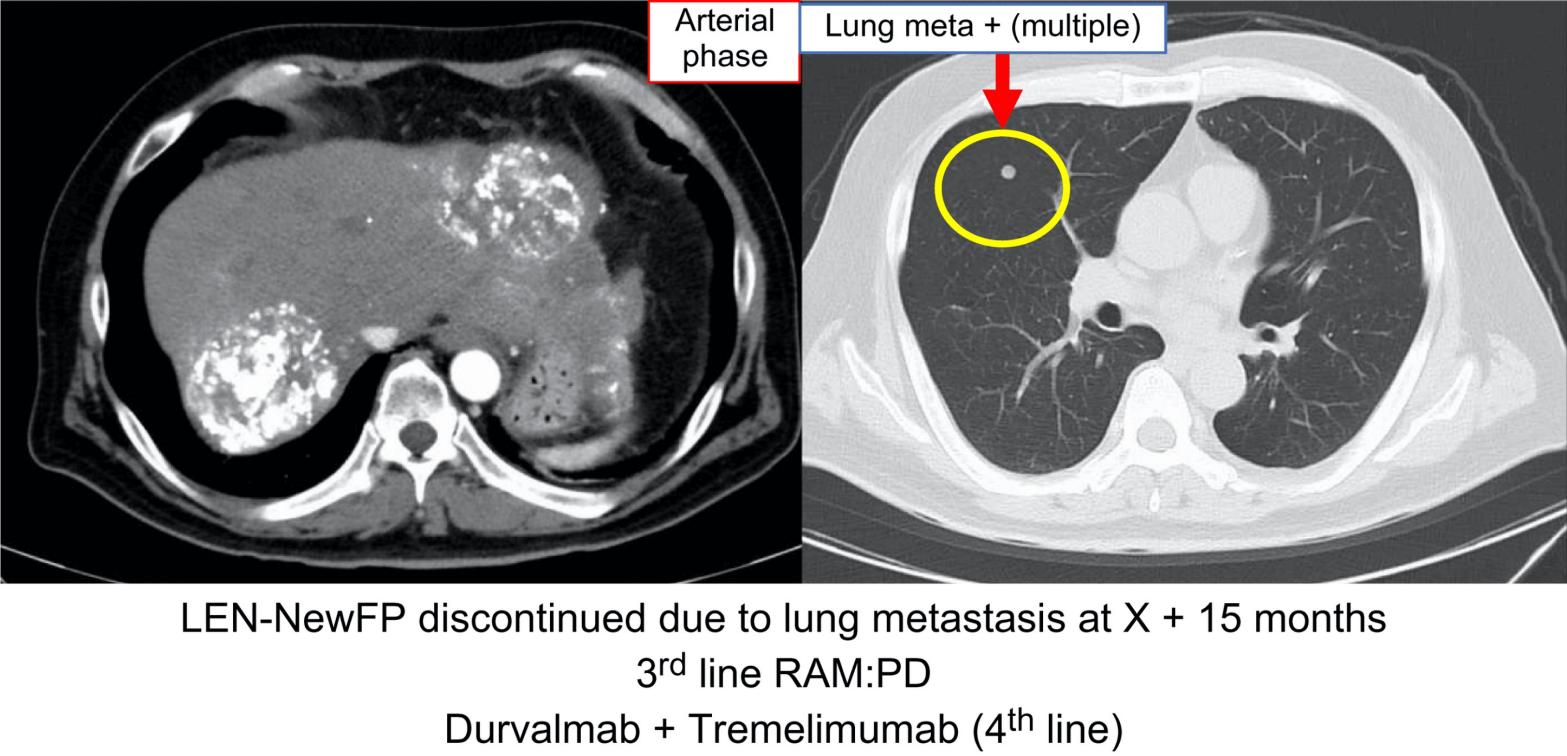
Contrast-enhanced CT on X+2 February showed good intrahepatic disease control and no intrahepatic viable tumor; however, a new pulmonary metastasis was detected. LEN: lenvatinib; RAM: ramucirumab; PD: progressive disease

**Figure 9:**
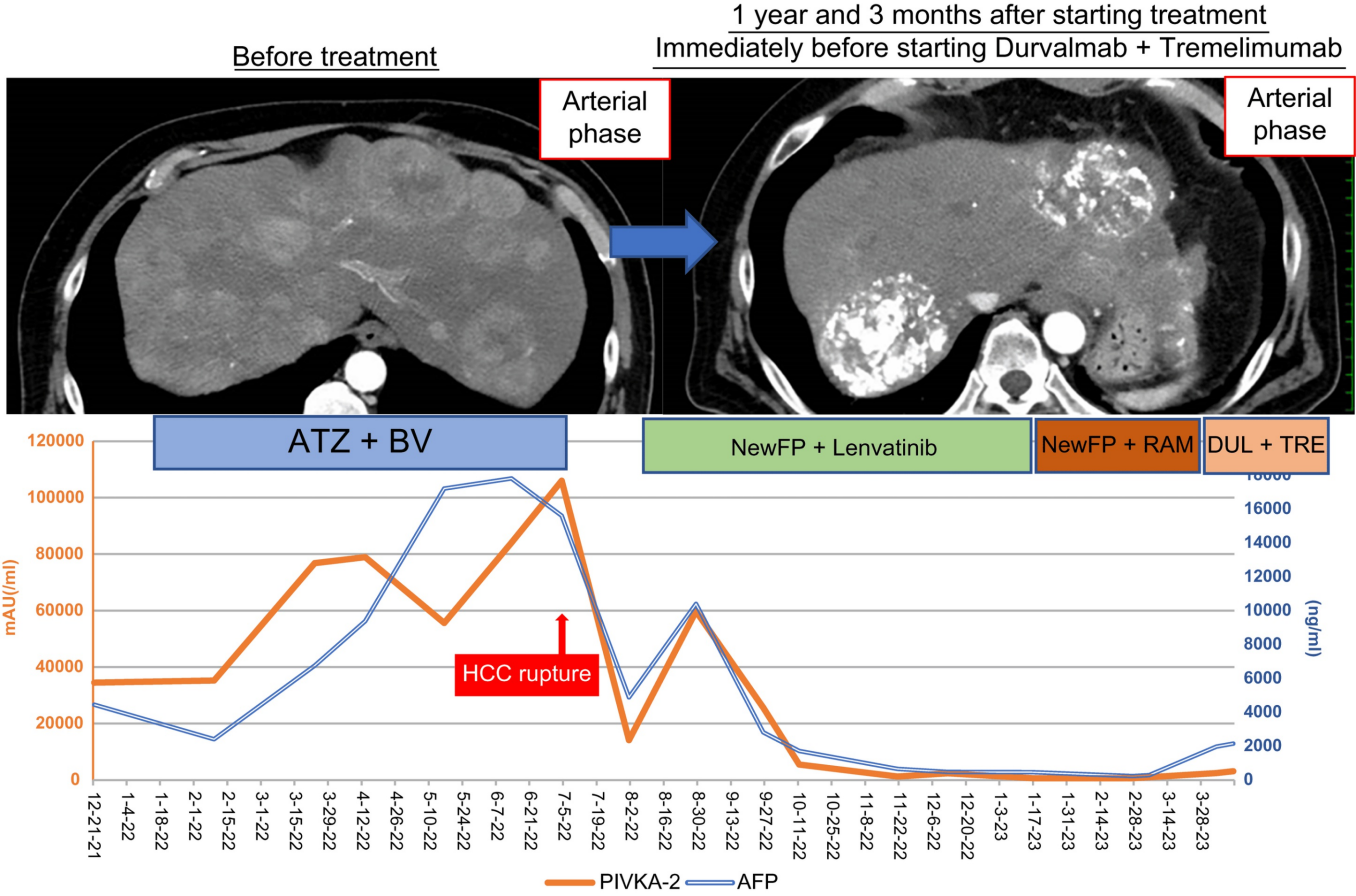
The clinical course from the start of atezolizumab+bevacizumab through LEN+New FP to durvalumab+tremelimumab administration is shown. ATZ: atezolizumab; BV: bevacizumab; LEN: lenvatinib; HAIC: hepatic arterial infusion chemotherapy; RAM: ramucirumab; DUL: durvalumab; TRE: tremelimumab

**Figure 10:**
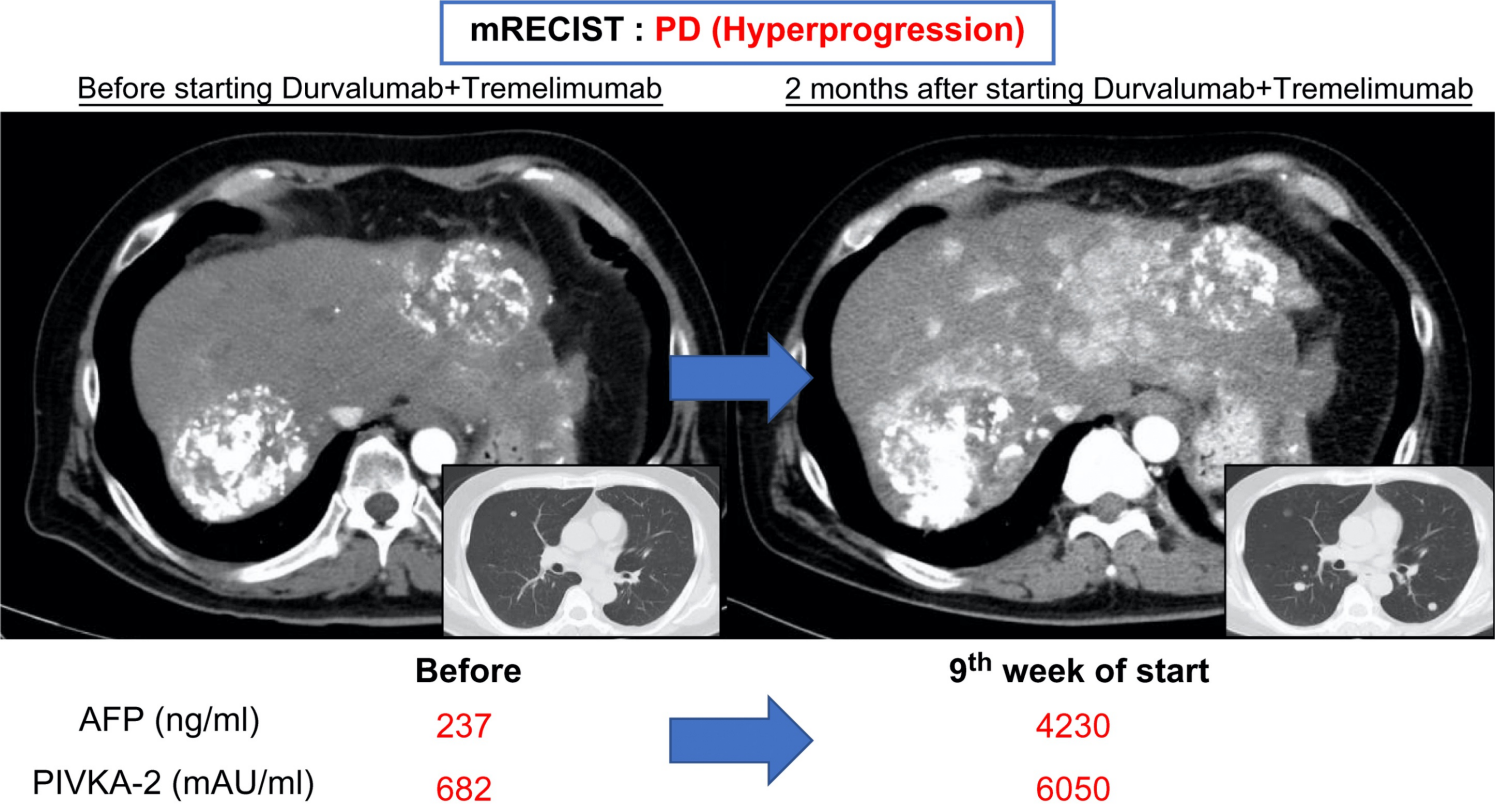
The patient was diagnosed with PD (hyperprogression) two months after starting durvalumab plus tremelimumab. mRECIST: modified Response Evaluation Criteria in Solid Tumors; PD: progressive disease; AFP: alpha-fetoprotein; PIVKA-2: protein induced by vitamin K absence or antagonist-II

However, in July 2022, the patient presented to the emergency department with epigastric pain and shock. A contrast-enhanced CT scan identified a hematoma formation around the HCC in liver segment S2, resulting in the detection of the ruptured HCC (Figure 3). Emergency transcatheter arterial embolization was performed, and extravasation of the contrast agent was identified in A2. Selective embolization was performed using lipiodol and a gelatin sponge (Serescue, Astellas Pharma Inc., Tokyo, Japan), and hemostasis was achieved. After the patient's overall condition stabilized, the contrast-enhanced CT was re-examined. Although extensive tumor necrosis was newly observed owing to hemorrhagic shock caused by the rupture of the HCC, most of the multiple HCCs in both lobes remained, while the portal vein tumor thrombus had progressed to Vp4 (Figure 4). Progressive disease (PD) was determined based on the course of the ruptured HCC and CT imaging evaluation. We administered the New FP regimen via HAIC in August 2022 owing to tumor invasion into the main portal vein. After two courses of New FP, contrast-enhanced CT evaluation showed increased target lesions, the appearance of new intrahepatic lesions, and a trend toward increased tumor markers; however, the disease was determined to be PD (Figure 5). Consequently, in September 2022, we combined oral lenvatinib with the third course of New FP which resulted in a significant decrease in tumor markers. Contrast-enhanced CT in December 2022 revealed CR according to mRECIST at the target lesion (Figure 6); however, intrahepatic tumor recurrence was noted in the S6 and S2 liver regions (Figure 7). TACE plus stereotactic body radiotherapy was applied to these regions for local control because the S6 tumor recurred in the right inferior hepatic artery and the S2 tumor recurred in the left inferior hepatic artery and extrahepatic feeder tract. Following this, the disease was assessed as PD, leading to a switch to New FP+ramucirumab. Contrast-enhanced CT in February 2023 demonstrated effective intrahepatic disease control with no intrahepatic viable tumor; however, a new pulmonary metastasis was identified (Figure 8), indicating PD. By April 2023, HAIC+ramucirumab treatment was discontinued in favor of durvalumab+tremelimumab (Figure 9). However, contrast-enhanced CT for efficacy evaluation revealed significant progression of HCC recurrence and a marked increase in tumor markers, indicating PD (hyperprogression) (Figure 10), leading to the cessation of durvalumab+tremelimumab administration. Subsequently, the focus was shifted towards intrahepatic tumor control, resuming LEN-New FP administration. The patient is currently undergoing outpatient treatment with LEN-New FP.

## Discussion

In this study, based on the favorable results of the LEOPARD trial, we hypothesized that adding lenvatinib to New FP instead of intraarterial cisplatin injection would provide better antitumor effects. Consequently, we encountered an interesting case with a clinical course, suggesting the possibility of a synergistic effect between lenvatinib and New FP. Additionally, when we pooled the treated cases, we obtained response and disease control rates that exceeded those of the LEOPARD study. Moreover, all patients had a history of prior administration of atezolizumab+bevacizumab, suggesting its usefulness as a treatment option after second- or third-line treatment of advanced HCC.

In the presented case, the initial treatment evaluation with New FP monotherapy was PD; however, since the patient exhibited factors for poor OS owing to New FP monotherapy, according to Niizeki et al., the course was considered to be consistent with previous studies [11,13]. Nevertheless, intrahepatic recurrence of two lesions occurred simultaneously with lung metastasis despite LEN-New FP administration. Moreover, the feeders were the right renal capsular artery and left inferior phrenic artery, and there was no intrahepatic recurrence that affected HAIC treatment. This implies that although PD was observed in organs other than the liver, such as the lungs, where only lenvatinib alone can be effective, the disease was controlled within the liver, which is the therapeutic area of ​​LEN-New FP. Furthermore, when New FP monotherapy was administered, PD was observed even within the liver. This suggests that LEN-New FP had a synergistic effect, resulting in a high therapeutic effect.

In addition, compared to the LEOPARD trial, New FP therapy contains lipiodol, which has a weak embolic effect and the ability to remain in the tumor [21]. Therefore, it has a synergistic effect with lenvatinib's reduction of the tumor vascular bed, making it even more effective. We speculate that this will lead to high antitumor effects. In recent years, the usefulness of lenvatinib [22] and lenvatinib+one-shot HAIC [23] has been reported in patients who are unresponsive to atezolizumab+bevacizumab. However, no study has reported the use of LEN-New FP after ICI failure. Although it is necessary to compare cases wherein ICI was used and not used, our study suggests that LEN-New FP may be a potential treatment option after atezolizumab+bevacizumab refractoriness.

Furthermore, in recent years, several studies have shown the usefulness of LEN-TACE for HCC; therefore, the combination of lenvatinib and endovascular treatment can be considered a trend in HCC treatment. Since LEN-New FP is also an endovascular treatment, a debate regarding the appropriate treatment choice between LEN-TACE and LEN-New FP is expected. Therefore, we compared the target patient groups for LEN-TACE in previous studies to the target patient group for LEN-New FP in the present study. In the TACTICS-L trial [14], the inclusion criteria for LEN-TACE were restrictive: tumor diameter ≤10 cm, number of tumors ≤10, vascular invasion, and extrahepatic metastasis were excluded from cases. Similarly, in the LAUNCH trial [15], a clinical trial of LEN-TACE conducted in China, tumors with one or more measurable lesions in the liver, one lesion <10 cm, or <10 lesions with a tumor burden of <50% were included. This is thought to be due to the selection of a group of tumor conditions that do not significantly deviate from the Up-to-7 criteria, which has recently been considered a positive indication for TACE, when conducting clinical trials.

In our study, treatment with LEN-New FP achieved a high response rate and disease control rate, even though five out of six patients corresponded to the exclusion criteria of the TACTICS-L and LAUNCH trials. This suggests the need to reconsider eligible patients for LEN-TACE and LEN-New FP. The advantages of LEN-New FP are that it can be performed on an outpatient basis, is less expensive than TACE, can be easily repeated, and is effective even when LEN-TACE is unsuitable. Moreover, although lenvatinib plus repeated TACE or transcatheter arterial infusion (TAI) therapy can also provide high therapeutic efficacy, it is considered unsuitable for frequent short-term treatment because it does not provide the added benefit of 5-FU infusion and requires hospitalization each time, which is a significant physical, financial, and time burden for patients. Additionally, several evidence-based studies in recent years have evaluated the comparative safety and efficacy of TACE versus HAIC for unresectable HCC [17,18]. Despite the use of different HAIC regimens, HAIC likely provides better effects and survival benefits than a standard TACE treatment [17,18]. Hence, HAIC may be a more promising approach than TACE for patients with unresectable HCC.

Despite its advantages, this study had some limitations including the small number of registered patients, bias in background liver, sex, and tumor status owing to the small number of cases, and the fact that it was only reported by a single physician at a single institution. Therefore, the level of evidence is limited. Furthermore, all cases had a history of prior ICI use, and we cannot deny the possibility that lenvatinib and HAIC promoted the release of tumor antigens, creating a pseudo-combined state of ICIs remaining in the body and promoting antitumor effects. Therefore, future studies with larger sample sizes are required. Additionally, they should examine the differences in treatment effects depending on the presence or absence of a history of prior ICI administration. However, ICI is the first choice for the treatment of advanced HCC worldwide, and not administering ICI may have ethical problems.

## Conclusions

LEN-New FP, a combination of lenvatinib and New FP for advanced HCC, demonstrated a high response rate and proved more effective in high-risk patients with factors associated with poor OS compared to conventional New FP monotherapy. Additionally, LEN-New FP exhibited extremely high objective response and disease control rates and was well tolerated, even in cases where it is considered second- or third-line systemic chemotherapy for advanced HCC.

Although there has been a long-standing debate regarding atezolizumab+bevacizumab and post-lenvatinib treatment for advanced HCC, no definitive treatment strategy has been established yet; however, LEN-New FP may become a potential treatment option. Thus, LEN-New FP holds the potential to be a breakthrough therapy with judicious case selection. However, it is essential to accumulate additional data to establish evidence.
